# Anthropomorphizing Technology: A Conceptual Review of Anthropomorphism Research and How it Relates to Children’s Engagements with Digital Voice Assistants

**DOI:** 10.1007/s12124-021-09668-y

**Published:** 2021-11-23

**Authors:** Janik Festerling, Iram Siraj

**Affiliations:** Department of Education, University of Oxford, 15 Norham Gardens, Oxford, OX2 6PY UK

**Keywords:** Alexa, Anthropomorphism, Child development, Google Assistant, Human-computer interaction, Pragmatism, Social cognition, Voice assistants

## Abstract

‘Anthropomorphism’ is a popular term in the literature on human-technology engagements, in general, and child-technology engagements, in particular. But what does it really mean to ‘anthropomorphize’ something in today’s world? This conceptual review article, addressed to researchers interested in anthropomorphism and adjacent areas, reviews contemporary anthropomorphism research, and it offers a critical perspective on how anthropomorphism research relates to today’s children who grow up amid increasingly intelligent and omnipresent technologies, particularly digital voice assistants (e.g., Alexa, Google Assistant, Siri). First, the article reviews a comprehensive body of quantitative as well as qualitative anthropomorphism research and considers it within three different research perspectives: *descriptive*, *normative* and *explanatory*. Following a brief excursus on philosophical pragmatism, the article then discusses each research perspective from a pragmatistic viewpoint, with a special emphasis on child-technology and child-voice-assistant engagements, and it also challenges some popular notions in the literature. These notions include descriptive ‘as if’ parallels (e.g., child behaves ‘as if’ Alexa was a friend), or normative assumptions that human-human engagements are generally superior to human-technology engagements. Instead, the article reviews different examples from the literature suggesting the nature of anthropomorphism may change as humans’ experiential understandings of humanness change, and this may particularly apply to today’s children as their social cognition develops in interaction with technological entities which are increasingly characterized by unprecedented combinations of human and non-human qualities.

**Supplementary Information:**

The online version contains supplementary material available at 10.1007/s12124-021-09668-y.

## Introduction

Through natural learning, humans develop understandings of their increasingly technologized environments long before they attend school (Papert, 1980). In this context, the earliest sphere of human development, the family home, sets the first primary stage for children’s learning experiences, before their horizons begin to widen when they enter other childhood environments including their neighborhoods, pre-schools, primary schools or other family homes (Huston & Ripke, 2006). Within these broader home and childhood environments, a global socio-technical change has occurred over the last decade through the rise of commercially available Digital Voice Assistants (DVAs) like Amazon’s ‘Alexa’, Apple’s ‘Siri’, or Google’s ‘Google Assistant’ (Ammari et al., 2019; Beirl et al., 2019; Porcheron et al., 2018; Sciuto et al., 2018; Voit et al., 2020; Yuan et al., 2019; Beneteau et al., 2019; Festerling & Siraj, 2020; Gao et al., 2018; Garg & Sengupta, 2020; Lee et al., 2020; Lopatovska & Williams, 2018; Lovato et al., 2019; Lovato & Piper, 2015)*.* One-third of US adults already own stand-alone DVA-devices (e.g., smart speakers (Kinsella & Mutchler, 2020)), while forecasts predict the total number of DVA-enabled devices, which had already exceeded four billion in late 2020, will outnumber the human world population by 2024 (Moar & Escherich, 2020).

Although DVAs may neither be the only nor the most sophisticated manifestations of intelligently behaving technologies, these individually and communally accessible voice interfaces are one of the most obvious experiences of artificial intelligence in today’s home and childhood environments (Vlahos, 2019; Turk, 2016; Hoy, 2018; Hirschberg & Manning, 2015). In contrast to smart toys and other technologies solely designed for children, DVAs’ commercial ecosystems comprise a growing number of hardware (e.g., smart speakers, smart TVs, smartphones, smart home appliances, wearables, car entertainment systems) and software components (e.g., native and third-party DVA skills, professional/educational/medical DVA applications), which together are becoming *omnipresent* parts of everyday life (Small et al., 2018; Sweeney & Davis, 2020; Trippas et al., 2019; Trivedi, 2018; Wang et al., 2019a; Alagha & Helbing, 2019; Dousay & Hall, 2018; Jargon, 2020; Perez, 2019; Pradhan et al., 2019; Ross, 2019; Sezgin et al., 2020; Skidmore & Moore, 2019). In addition, preliminary research suggests DVAs’ omnipresence across various devices at home is especially appreciated by families (Meurisch et al., 2020), further underpinning the relevance of DVAs in the context of developmental research.

But it is not only the growing socio-technical omnipresence of DVAs which could make them an important case for developmental research, but also their potential ontological momentum as experienced by today’s children. DVAs are part of the human-technology dyad which has evolved since humans first used stones to break open coconuts or fallen trees to bridge rivers (Harwood & Eaves, 2020). In this dyad, DVAs represent the stage of “autonomous technological beings” (Harwood & Eaves, 2020, p.7), which are able to emulate peculiar qualities of human beings like language and speech, and, as Nass & Brave (Nass & Brave, 2005, p.3 emphasis in original) point out, “over the course of 200,000 years of evolution, humans have become *voice-activated* with brains that are wired to equate voices with people and to act quickly on that identification”. However, it should also be remembered humans seem to have a natural tendency to ‘equate’ almost anything with people, and to act quickly on this identification through something formally known as *anthropomorphism*.

Anthropomorphism refers to the psychological phenomenon that humans tend to *engage* socially with non-human entities (e.g., technology, animals, plants, supranatural entities, natural or social phenomena) as if these entities were human. These humanlike engagements include their *behaviors* (i.e., how humans interact with non-human entities), their *feelings* (i.e., how humans feel about non-human entities) and their *perceptions* (i.e., how humans see and think about non-human entities) (Epley et al., 2007).^1^ This phenomenon is seen across various societal, cultural, religious and historical contexts (Guthrie, 1993), and has been scrutinized across diverse fields of research including psychology, neuroscience, psychiatry, philosophy, ethology and education (Varella, 2018). But since the growing digitization of the environment began in the second half of the twentieth century, anthropomorphism has become particularly relevant in addressing how humans engage socially with technology – with personal computers, basic robots, electronic toys etc. (e.g., (Reeves & Nass, 1996; Turkle, 1984/2005)). With ongoing technological progress (e.g., smartphones, robotics, autonomous driving), anthropomorphism continues to be a popular research theme in the literature (e.g., (Turkle, 2017; van Straten et al., 2020; Wang, 2017; Waytz et al., 2014a)), and especially in the context of DVAs (e.g., (Voit et al., 2020; Gao et al., 2018; Lee et al., 2020; Lopatovska & Williams, 2018; Pradhan et al., 2019; Biele et al., 2019; Motalebi et al., 2019; Purington et al., 2017; Wagner & Schramm-Klein, 2019)).

Despite this wealth of material, little attention has been paid to the systematic differences between commercial DVAs and the animal-like or human-like technologies (e.g., *Aibo*, *Robovie*, *Pepper*, *Nao*, *Kismet, iCat*) and toys (e.g., *Tamagotchi*, *Furby*) often used in anthropomorphism research (Festerling & Siraj, 2020). For example, most of these robotic technologies and toys convey the notion of being self-contained entities (i.e., entities which engage with their environments based on direct sensory input through cameras, microphones, or sensors, and which seem intelligent in the sense that they can somehow process this input internally). In contrast, children may experience DVAs as a different phenomenon, because they appear both to be omnipresent in various environments at the same time and to be intelligent in the sense that they are closely intertwined with sources above and beyond their physical boundaries (e.g., internet, smart home sensors). Furthermore, robotic technologies and toys are often designed to convey strong visual and haptic experiences (e.g., human- or animal-like embodiments through eyes, mouths, ears, arms or legs), or to evoke caring or nurturing behaviors (e.g., feeding). As a consequence, such entities are usually limited to specific age ranges, and children eventually grow out of playing with them (Festerling & Siraj, 2020). In contrast, DVAs are not limited to specific age ranges: their minimalistic physical designs (e.g., smart speakers) and utilitarian ‘personalities’ with a constantly growing number of functionalities and applications constitute an appeal to infants as well as elderly people, therefore turning DVAs into potential lifetime companions in their own right (Festerling & Siraj, 2020). In the context of daily family life, this could mean that children rarely observe adults engaging socially with toys, yet they may often observe adults engaging socially with DVAs through human language and speech, therefore prompting children to conclude DVAs are serious voice-enabled engagement partners in their own right. Lastly, it should also be noted that although DVAs may be *less* embodied in terms of animal-like or human-like appearances (e.g., compared to robots), DVAs’ are still physically embodied entities visually conveying socially salient cues (e.g., user-oriented display movement, pulsing light ring).^2^

The key question remains how anthropomorphism research relates to children who grow up amid increasingly intelligent and omnipresent technologies, particularly DVAs. This article suggests the answer may be less obvious than it seems. In particular, it is argued the nature of anthropomorphism may change as humans’ experiential understandings of humanness change, and this may particularly apply to today’s children who grow up amid increasingly intelligent and omnipresent technologies, such as DVAs. This article uses the methodological basis of a conceptual review, which means the synthesis of the interdisciplinary body of literature follows a conceptually framed process to address the key question of interest (Ayala, 2018; Parsons, 2016). The process is as follows: the first section reviews anthropomorphism research according to three different research perspectives (see research overview in supplementary material). Following a brief excursus on philosophical pragmatism, the second section then assesses how each of these perspectives relates to children who grow up amid increasingly intelligent and omnipresent technologies, such as DVAs. Finally, the last section summarizes the main arguments, outlines its limitations, and offers some directions for future research.

## *What Is it? What Are its Consequences? What’s Causing it?* A Triadic View on Anthropomorphism Research

Inspired by Epley’s (2018) core questions which anthropomorphism research should address (e.g., ‘What is it?’, ‘What are its consequences?’, ‘What’s causing it?’), this section reviews anthropomorphism research according to three different research perspectives. The purpose of this triadic view on anthropomorphism research is to provide a comprehensive account of contemporary anthropomorphism research in the context of human-technology engagements. Facing the trade-off between analytical scope and depth, the following review refers to ‘technology’ and ‘social engagements’ as rather broad categories comprising various types of technological entities and engagements (see research overview in supplementary material), while special attention is only paid to the peculiarities of children’s engagements with DVAs. Although this is not without limitations, taking this approach is in line with the review’s overall objective to look at the bigger picture of contemporary research investigating humans’ engagements with technology, in general, and children’s engagements with technology, in particular.

### *What Is it?* Anthropomorphism from a *Descriptive* Research Perspective

From a *descriptive* perspective, anthropomorphism research uses human-human engagements as a benchmark to investigate empirically observable parallels to human-technology engagements. This is, to what extent humans seem to engage with non-human technology *as if* these technological entities were human beings.

Early empirical work which suggested human engagements with technology bear deep parallels with human-human engagement patterns (e.g., (Reeves & Nass, 1996; Nass et al., 1993, 1994, 1996, 1999)) has been followed by an array of research on the different delineations and dependencies of anthropomorphism (for more details see research overview in supplementary material). For example:

- more humanlike appearances and behaviors of technological entities can directly increase how humans rate the entities’ human-likeness and likability (e.g., (DiSalvo et al., 2002; Walters et al., 2008));
- more humanlike technological entities can convey signals of ‘intention’ (e.g., gazing) better to humans compared to less humanlike technological entities (e.g., (Mutlu et al., 2009));
- humans tend to engage in less abusive behaviors towards technological entities when the entities appear more intelligent (e.g., (Bartneck & Hu, 2008));
- humans’ trust in humanlike technological entities can be more resilient compared to their trust in less humanlike technological entities (e.g., (de Visser et al., 2016));
- voice-enabled technological entities can be held more morally accountable for their actions than simple technological entities without voice-interfaces (e.g., (Kahn et al., 2012a));
- technological entities with non-verbal emotional expressions, or with humanlike rather than robotic voices, can yield higher levels of acceptance and attachments in humans (e.g., (Eyssel et al., 2010, 2012)).

With regard to DVAs, humans who personify these voice-enabled technologies (e.g., referring to DVA with personal pronouns) tend to be more satisfied with their overall engagement experiences, and tend to cultivate more sociable relationships with them (Purington et al., 2017). Additionally, DVAs which emulate humanlike empathetic responses after being insulted (e.g., ‘You’re upset, let me know if there’s something I can do better’) can trigger feelings of guilt or mitigate aggressiveness in humans (Chin et al., 2020).

This suggests anthropomorphism requires the presence of humanlike ‘triggers’ in the environment (Waytz et al., 2014b, 2019), yet empirical research has shown humanlike engagement patterns with technological entities also depend on humans themselves. The patterns depend, for example:

- on humans’ own gender or ethnicity, and how it matches technology’s embodied gender or ethnicity (e.g., (Eyssel et al., 2012; Kamide et al., 2013));
- on humans’ personalities (e.g., (Walters et al., 2008));
- on humans’ socio-cultural backgrounds (e.g., (Evers et al., 2008));
- on humans’ prior experience with technology (e.g., (Goudey & Bonnin, 2016));
- on humans’ needs and feelings for sociality or control (e.g., (Wang, 2017; Eyssel & Reich, 2013; Kim & McGill, 2011; Waytz et al., 2010a));
- on humans’ age (e.g., (Kamide et al., 2013)).

However, the role of age in anthropomorphism remains intricate, or, as (Airenti, 2018, p.11) states, “the difference between adults and children is not qualitative but rather a matter of complexity”. For example, although infants and young children often engage socially with non-human entities (e.g., toys) by *pretending* the entities are human (Airenti, 2015a), they also possess firm understandings of the ontological borders they cross and the behavioral commitments they make (e.g., imagination vs. reality) (Harris, 2000). This suggests children’s anthropomorphism (as with adults’) cannot be reduced to naïve confusions or infantile pretend play (Airenti, 2018; Severson & Woodard, 2018; Severson & Lemm, 2016).

Despite this considerable research about the nature of anthropomorphism in human-technology engagements, the “promiscuous use of the term” (Epley, 2018, p.594) in the literature suggests there is still little consensus about a precise definition of *what* anthropomorphism *is* and *is not*. Although the attribution of mental states (e.g., agency, motivations, interests, emotions, knowledge, sociality, moral worth and responsibility) lies at the heart of anthropomorphism for many authors (e.g., (Epley et al., 2007; Severson & Lemm, 2016; Caporael & Heyes, 1997; Fisher, 1996; Reiss, 2017; Severson & Carlson, 2010; Urquiza-Haas & Kotrschal, 2015)), other empirical studies suggest anthropomorphism it is not merely about the attribution of mental states; rather, it is about the holistic ontological concept of *humanness* people apply when engaging socially with non-human entities (Shaman et al., 2018). However, these underlying concepts of anthropomorphism (humanness vs. non-humanness) often seem too intricate to explicate, yet are appealingly intuitive to human audiences (Varella, 2018). It is, therefore, unsurprising that anthropomorphism research continues to explore almost any corner of the social experiences which humans can potentially have with other humans. This suggests the alleged ‘promiscuity’ more likely reflects what anthropomorphism research is about from a descriptive perspective: investigating parallels between the empirically observable patterns of human-technology engagements and the openly interpretable benchmark line of human-human engagements. Yet investigating parallelisms does not require consensus on the ‘true’ nature of the designated benchmark line (i.e., ‘true’ nature of human-human engagements), it only requires minimal consensus on what its designated endpoints are (i.e., human-human) in order to identify parallels between alternate endpoints (e.g., human-technology) – and there is, of course, little disagreement about who these living organisms, widely referred to as ‘humans’, actually are.

### *What Are its Consequences?* Anthropomorphism from a *Normative* Research Perspective

As expressed by Epley (2018, p.695), something “that has no demonstrable consequences is not worth studying”. The above discussion already suggests anthropomorphism has various consequences for human-technology engagements, but, from a *normative* perspective, the important question is how to evaluate such empirically delineated consequences.

On the one hand, these evaluations can yield concrete recommendations about how to improve technology’s usability and effectiveness in human-technology engagements through the nuances of humanlike designs (e.g., (Waytz et al., 2014a; Breazeal, 2002; Duffy, 2003; Fong et al., 2003; Kiesler & Hinds, 2004; Norman, 2005; Schmitz, 2010; Triberti et al., 2017; Waytz et al., 2010b)). Therefore, from a normative perspective, anthropomorphism research offers various potentials and opportunities to exploit the human tendency to engage socially with technological entities for meaningful and practical purposes. This is sometimes referred to as ‘applied anthropomorphism’ (Damiano & Dumouchel, 2018). For example:

- naturalistic and humanlike movements can enhance technology’s likeability (e.g., (Castro-González et al., 2016));
- technological entities endowed with conversational humanlike design components (e.g., microphones which look like ears; speakers which look like mouths) can give human users an immediate intuition of how to engage with them (e.g., (Złotowski et al., 2015));
- more humanlike technological entities can facilitate the collaborative effectiveness of human-technology engagements, or they can evoke socially desirable behaviors from humans – like refraining from harming the entity (e.g., (Bartneck & Hu, 2008; Shah et al., 2011; Złotowski et al., 2014));
- engaging with humanlike technologies can serve as a ‘scaffold’ for children with autism to improve their social cognition (e.g., (Atherton & Cross, 2018)).

But, evidently, whether the integration of humanlike design components is desirable also depends on the concrete applications for which technological entities are used (e.g., (Złotowski et al., 2015; Choi & Kim, 2009; Collins, 2017; Goetz et al., 2003; Riether et al., 2012)), especially in the context of child-technology engagements (Pearson & Borenstein, 2014). For example, although social robots have proven their promising potential as learning technologies (for reviews see (Belpaeme et al., 2018; Kanero et al., 2018; Papadopoulos et al., 2020)) – and this seems to hold true for DVAs as well (e.g., (Xu et al., 2021)) – children can also develop adverse responses to too much human-likeness (e.g., (Brink et al., 2019; Woods, 2006; Yip et al., 2019)), therefore echoing Mori’s (Mori, 2012) widely-cited ‘uncanny valley’ theorem about the eeriness of *almost* perfect human resemblance.

On the other hand, the consequences of anthropomorphism in human-technology engagements have prompted researchers to evaluate the ethical risks of endowing technology with humanlike design components (Złotowski et al., 2015; de Graaf, 2016; Sætra, 2020, 2021a, 2021b). At their core, such ethical criticisms, sometimes referred to as the ‘forensic problem of anthropomorphism’ (Złotowski et al., 2015), consider the creation of humanlike technology as a form of deception, because they ‘trick’ humans, especially children, into overestimating the technology’s ‘true’ capacities (Sharkey & Sharkey, 2010), or into engaging with the technology in ways which are supposed to be reserved for genuine human-human engagements (Sætra, 2020). This may even culminate in the fear that humanlike technology could constitute a threat to human distinctiveness (e.g., (Ferrari et al., 2016; Porra et al., 2020)). At the forefront of this criticism, Turkle (1984/2005, 2017) has repeatedly emphasized how the temptations of engaging with technology, in general, and humanlike technology, in particular, appeal to human *vulnerabilities* rather than human *needs*, because the technological entities in question constitute seductive appeals to replace, denigrate, deny or degrade what deserves integrity: the existential value of human-human engagements. According to this normative perspective, no matter how authentic a technology’s embodied human-likeness becomes in terms of its experienceable social qualities, and no matter how much humans appreciate and enjoy engaging socially with this particular technology, there remains an unambiguous normative hierarchy between engaging with ‘simulations’ of human nature and with human nature itself (Turkle, 1984/2005, 2017; Sætra, 2020). More recently, Turkle (2018) has re-emphasized this normative positioning in the context of DVAs by arguing children who engage with DVAs, and who may even develop social bonds or a sense of friendship with them, are ultimately at risk of ‘forgetting what it means to be human’.

### *What’s Causing it?* Anthropomorphism from an *Explanatory* Research Perspective

At its core, anthropomorphism is a psychological phenomenon, and, as such, the causes of its origins and variability must be explainable by psychological mechanisms (Epley, 2018). From an explanatory research perspective, this means there must be a psychological reasoning for the question *why* humans tend to engage socially with non-human technology as if these entities were humans. The answer lies in the heart of human social cognition.

Social cognition seems to be a particularly strong quality of human intelligence compared to other primate species (Herrmann et al., 2007), and there are good reasons to assume the foundations of social cognition are innate due to their early manifestations in human development (e.g., (Airenti, 2015b; Kovacs et al., 2010; Onishi & Baillargeon, 2005; Perner & Roessler, 2012; Southgate et al., 2007)). As humans develop, social cognition culminates, among other things, in the ability to infer and contemplate the intricate perspectives of others in their beliefs, interests and motivations for action – usually referred to as ‘Theory of Mind’ (ToM) (Wellman, 2014). But what about non-human others?

Social cognition is a *pervasive* psychological ability, and, consequently, anthropomorphism should not be reduced to “a by-product of misplaced social cognition […]; rather [it is] an unavoidable consequence of the functional organization of the human brain” (Urquiza-Haas & Kotrschal, 2015, p.171). For example, children’s early ability to evaluate the passively observed social behaviors of others (Hamlin et al., 2007) seems to extend naturally to human-technology engagements, as exemplified by 18-month-old infants who can already recognize whether a robot engages in socially contingent dialogues with adults (Meltzoff et al., 2010). Furthermore, neuroscientific evidence suggests the same neural mechanisms of social cognition (e.g., attribution of mental states to others, responding to facial expressions) are also at work when humans anthropomorphize non-human entities (e.g., (Castelli et al., 2000; Chaminade et al., 2007; Cullen et al., 2014; Dubal et al., 2011; Gazzola et al., 2007; Gobbini et al., 2010; Scheele et al., 2015)).

From an evolutionary perspective, this propensity of social cognition to be triggered by human and non-human entities can be explained as an adaptive survival mechanism. In primeval environments, which had the greatest evolutionary impact on the development of the ‘modern’ human brain (Cosmides et al., 1992), it was a more effective strategy to behave falsely as if something was alive and similar to oneself, and a more fatal strategy to behave falsely as if something was inanimate and without agency or intelligence (e.g., (Guthrie, 1993; Atran & Norenzayan, 2004)). Notably, even today individual tendencies to anthropomorphize non-human entities are positively associated with other evolutionary salient behaviors (e.g., hoarding) (Timpano & Shaw, 2013), and also other non-human primate species (e.g., chimpanzees) tend to engage socially with technological entities when the entities imitate the bodily movements of the animal engagement partner (Davila-Ross et al., 2014).

**Fig. 1:**
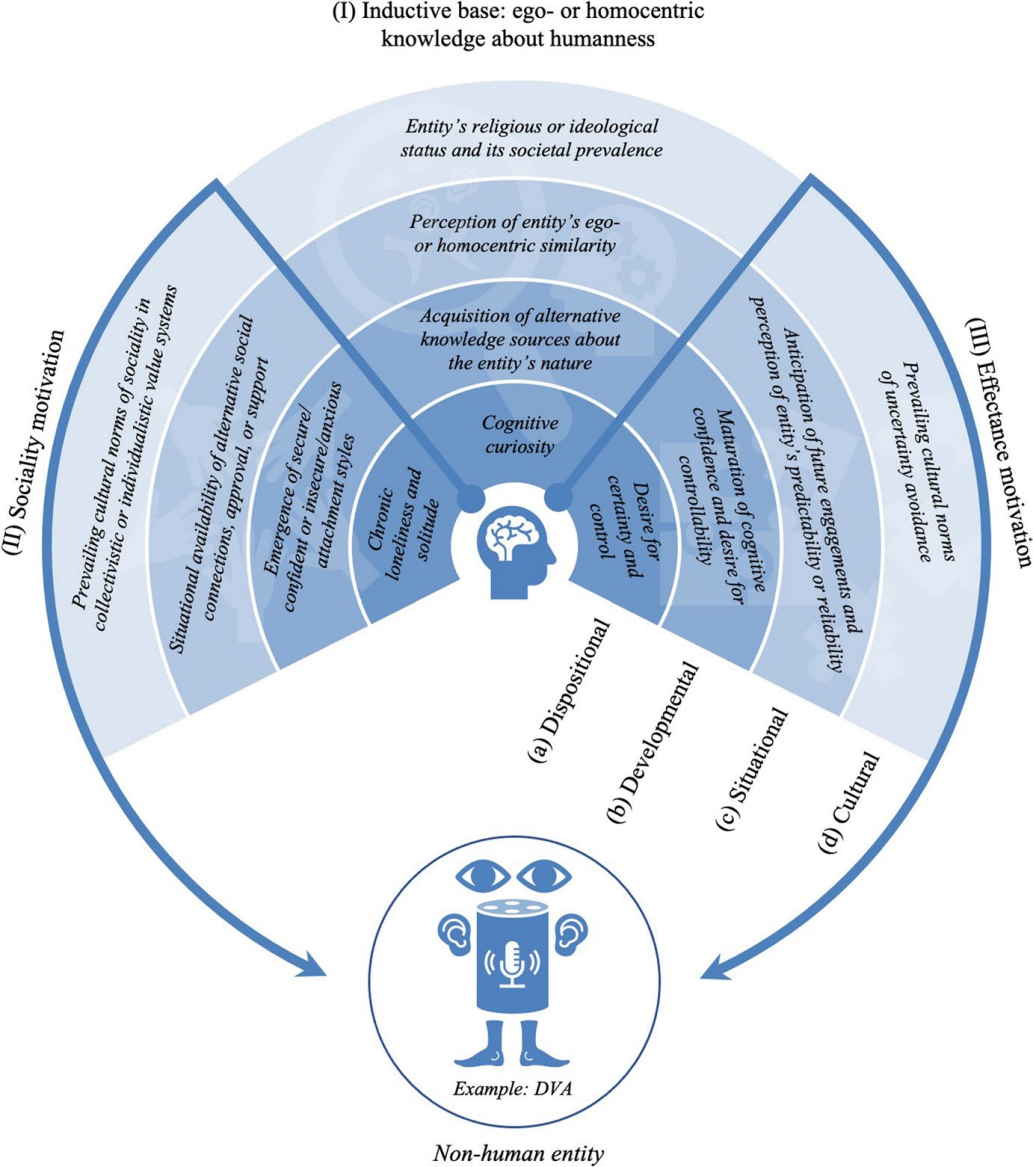
Determinants of anthropomorphism based on Epley et al.’s three-factor theory. *Notes.* Figure visualizes anthropomorphism according to Epley et al.’s (2007) three factor theory of anthropomorphism. Circular segments (I to III) visualize determinants of anthropomorphism: (I) inductive base of ego- or homocentric knowledge about humanness (cognitive determinant), (II) sociality motivation (first motivational co-determinant), and (III) effectance motivation (second motivational co-determinant). Different shades within each circular segment show examples for (a) dispositional, (b) developmental, (c) situational, and (d) cultural sub-determinants. Figure displays a DVA as an example of a non-human entity. Some key terms have been adjusted compared to Epley et al.’s (2007) original terminology (e.g., ‘need for cognition’ → ‘cognitive curiosity’). *Source.* Developed from Epley et al. (2007)

In line with this brief account of social cognition, Epley et al.’s (2007) widely-cited three-factor theory assumes anthropomorphizing non-human entities follows the same cognitive process of inductive inference which also orchestrates human-human engagements, namely *inferring* concrete assumptions and predictions about the otherwise opaque inner nature of social engagement partners. According to Epley et al. (2007), this inference is anchored in the inductive base of *humanness*, in humans’ deeply ingrained knowledge about the self in particular (i.e., direct phenomenological experience of being human resulting in *egocentric knowledge*), and about humans in general (i.e., experience-based knowledge of humans resulting in *homocentric knowledge*). Although it is challenging to disentangle the ego- and homocentric cognitive basis of anthropomorphism (see (Kwan et al., 2008)), it is important to note human social cognition offers different routes, ranging from using one’s own mind for simulating the mental processes of others (e.g., mirroring, self-projection), to using more abstract and knowledge-based forms of inference (e.g., mentalizing) (Van Overwalle & Baetens, 2009; Waytz & Mitchell, 2011). Furthermore, this readily accessible cognitive anchor of humanness is co-determined by two fundamental motivational forces, namely sociality motivation (i.e., human desire to seek social connections, approval, support etc.) and effectance motivation (i.e., human desire to gain efficacious cognitive control over one’s environment) (Epley et al., 2007). These motivational co-determinants are important factors in explaining the empirically observable variability of anthropomorphism across humans (e.g., (Wang, 2017; Waytz et al., 2010a; Epley et al., 2008)), and they can also account for the presence of anthropomorphism in populations with impaired social cognition (e.g., autism) (Atherton & Cross, 2018). However, the inductive base of humanness as the cognitive anchor of anthropomorphism, and its co-determinants of sociality and effectance motivation, are far from uniform, especially when comparing urban and rural populations (e.g., (Herrmann & Atricia, 2010; Medin et al., 2010)). Therefore, as outlined by Epley et al. (2007), the full variability of anthropomorphism can be explained only by taking into account the dispositional, developmental, situational and cultural sub-determinants of each factor (see Fig. 1).

## A Pragmatistic View on Anthropomorphism Research in the Context of Children and DVAs

The current understandings of anthropomorphism, as reviewed above, seem to assume the dualistic concepts of humanness and non-humanness, which define the existence of anthropomorphism, bear a metaphysical truth (i.e., represent true ontological delineations). But what if there is no metaphysical truth to such concepts? What if these concepts are epistemological instruments made up by the clever animals (i.e., humans) who use them? In other words, what if “truth, at its core, is not a metaphysical category but rather a moral and epistemological one” (Suckiel, 2006, p.37)? This is the provoking story of philosophical pragmatism, particularly one of its variants referred to as *instrumentalism* or *constructive empiricism *(Misak, 2006), which dates back to the classical pragmatist William James (e.g., (James, 1967, 1907/2010, 1912/2012)).^3^

Despite the different nuances which have evolved since its emergence in the late nineteenth century, classical pragmatism shares three philosophical themes: (1) the *active* and *subjective* role of the interpretative human mind in scientific and non-scientific knowledge acquisition; (2) the *fallible* and *non-apodictic* nature of knowledge due to its reliance on human experience; and (3) the rejection of *foundationalist* truths and certainties such as the Cartesian mind-body dualism (McDermid, 2006). These themes are certainly not exclusive to pragmatism per se, because as a rather heterogenous tradition, it has shares overlaps with other philosophical paradigms. For instance, pragmatism’s adherence to a mind-independent reality follows the rationale of realism. However, rather than assuming that realism allows us to get reality objectively ‘right’, it is considered a *hypothesis* we need to be able to learn by experience (McDermid, 2006; Rescher, 2006). For Jamesian pragmatism, this process of learning by experience is *teleological* in nature, meaning human cognition is always subject to individual motivations and interests (Suckiel, 2006). Therefore, beliefs about knowledge and truth must always be viewed in light of the believers’ motivations and interests, and, as James’ (1902/2013) work on religious experiences suggests, knowledge is true insofar as it has real consequences for those who believe in it.

From this pragmatistic viewpoint, the dualistic concepts of humanness and non-humanness are understood as functional distinctions for dealing with reality, and as true only insofar as they are practically useful for those who believe them according to their motivations and interests (Suckiel, 2006; Sprigge, 2006). In other words, pragmatism it is not repudiating dualistic concepts as such; rather, it is less concerned with their metaphysical validity and considers that “supposedly sharp distinctions may be better conceived as lines of demarcation drawn at some point on a continuum” (Haack, 2006, p.152). The question remains what a pragmatistic view on anthropomorphism research means in the context of children who grow up amid increasingly intelligent and omnipresent technologies, such as DVAs. Following Servais’ (2018) recent pragmatistic account of anthropomorphism research in the context human-animal engagements, the main starting point is to focus less on a priori certainties and to prioritize the nuances of human experiences, such as children’s subjective experiences when engaging with DVAs in their home and childhood environments. Based on the three research perspectives reviewed above, this is discussed in more detail in the following.

### Pragmatistic View on the *Descriptive* Research Perspective

From a descriptive perspective, anthropomorphism research uses human-human engagements as a benchmark to investigate the extent to which humans engage with non-human technological entities *as if* these entities were human beings, while there is still little consensus about what a definition of anthropomorphism should comprise, as reflected by the wide range of interpretations in the literature (Epley, 2018).

But is it feasible, from a pragmatistic viewpoint, to call for more clear-cut conceptual boundaries of anthropomorphism (see (Epley, 2018))? This intention seems noble, but the philosophical subtext of defining clear-cut boundaries would be that there were corresponding metaphysical categories of the human and the non-human. From a pragmatistic viewpoint, this would be an epistemological arrogation, because such boundaries represent only human-made lines drawn on some continuum for practical purposes and without any finality in meaning. This is partly reflected by the gradual decline of traditional anthropomorphism research which equates humanlike engagement patterns with genuinely ‘false’ behaviors or beliefs (e.g., behaving falsely as if something was human). For example, the recent anthropomorphism literature on human-animal engagements suggests “much of what has been considered as anthropomorphic interpretations may in fact do more justice to the mental states of other animals than was previously believed” (Urquiza-Haas & Kotrschal, 2015, p.168), such as ‘behaving as if dogs really understand what humans say’ (see (Andics et al., 2016)). However, should the arguments be different for human-technology engagements (as compared to human-animal engagements)? In other words, is it not possible to be more certain about the things which differentiate humans from the things they have created themselves (e.g., DVAs)? A pragmatistic answer would be to ask whether these are the most useful questions to ask for the investigation of human-technology engagements from a descriptive perspective. After all, it lies in the nature of a continuum that it is “constituted as much by *difference* as by *similarity*” (Alexander, 2006, p.189, emphasis added).

On the continuum of social engagements, human-technology engagements circumscribe one of the infinitesimal variations of engaging socially with one’s environment. Yet to be on this continuum in the first place is simply an all-or-nothing question, because, as Seibt (2017) points out, social engagements of *any* kind require a basic social commitment, which can either be undertaken or not, but it must always be *real.* In the words of Seibt (2017, pp.18–19, emphasis in original): “pretending to undertake a commitment is simply *to fail* to undertake it. […] Making a promise or treating someone as a person are real social interactions by *virtue* of engaging in a certain declarative practice”. On this basis, claiming children or adults engage socially with a technology ‘as if’ it was a human remains misleading: they either engage socially with it in one way or another, or they do not; but if they do, they have granted it the status of a social engagement partner in its own right. Moreover, and in line with the pragmatistic adherence to realism, Seibt (2017) emphasizes social engagements of *any* kind emerge from the reality of experienceable social behaviors as they are displayed by social engagements partners (e.g., facial expressions, language), regardless of whether these behaviors are deemed authentic or not. As Seibt (2017, pp.19–20, emphasis in original) writes:

[I]t is an obvious requirement of social praxis that the *performance conditions* of a social interaction […] must relate only to behavioral criteria and cannot take intentional states into account. […] [C]harges about someone’s performing a social action inauthentically or insincerely relate to the *quality* of the social action, not to [its] *occurrence*.

Both issues raised by Seibt (2017) seem particularly relevant in the context of DVAs. Alexa, the Google Assistant, Siri or other DVAs are designed to engage with humans through sophisticated social behaviors, namely the autonomous use of human language and speech (Festerling & Siraj, 2020). In light of such strong “behavioral realism” (Damiano & Dumouchel, 2018, p.3), the commitments of social engagement are supposedly easily undertaken (e.g., similar to the social behaviors displayed by animals) (Severson & Lemm, 2016), therefore granting DVAs the status of being social engagement partners in their own right – irrespective of whether their behaviors are perceived as being authentic or not. For empirical research, the pragmatistic viewpoint suggests children’s social engagements with DVAs reflect how they subjectively experience what they deem significant in terms of sociality, and that the nature of their engagements with DVAs reflects how they translate this experience of sociality into real social behaviors. Accordingly, the description that children engage with DVAs ‘as if’ these technological entities were friends, playmates, companions or simply humans, may not adequately capture what children subjectively experience.

In summary, narrowing the scope of anthropomorphism research by defining more precise conceptual boundaries is not a feasible endeavor. From a descriptive perspective, anthropomorphism research should continue to explore the infinitesimal variations of how children and adults engage socially with human or non-human entities in their environments, and to be informed or inspired by a general familiarity with human-human engagements – but not as an absolute and a priori defined benchmark.

### Pragmatistic View on the *Normative* Research Perspective

The above discussed pragmatistic view on anthropomorphism draws special attention to the normative issue of what humans deem worth engaging with, and whether granting the status of social engagement partners to technological entities (e.g., DVAs) could not only ennoble the technology but also degrade the human by deflating the unique social value of human-human engagements (e.g., (Turkle, 1984/2005, 2017)). However, can these concerns stand up to pragmatistic scrutiny?

The basic ethical criticism of endowing technology with humanlike design components is an inheritance from Descartes’ dualism (Descartes, 1649/1988, 1641/1998), especially regarding the strict ontological dichotomy of the mindful and the mindless, or the human and the non-human (Damiano & Dumouchel, 2018; Bruni et al., 2018). One of the most influential criticisms following this Cartesian legacy has been Turkle’s (1984/2005, 2017) hierarchical distinction between technology’s *inferior* simulation of mentation, intentionality or emotionality, and the *superior* genuineness of human mentation, intentionality or emotionality (Damiano & Dumouchel, 2018). Pragmatism is generally suspicious of drawing such apodictic conclusions from Cartesian a priori dualisms (McDermid, 2006), and it asks whether such an uncompromising hierarchization of superior human-human and inferior human-technology engagements can be justified by the normative virtues of human experience. This seems, at best, questionable, because such a hierarchization would ignore that, at times, humans could systemically prefer to engage with humanlike technology *due to,* rather than *despite of*, the absence of what they think constitutes ‘true’ humanness.

For example, there is comprehensive clinical research on how humans are more willing to self-disclose sensitive personal information to technology (e.g., human physician vs. virtual humanlike physician), and most findings suggest humans systematically prefer to self-disclose more sensitive personal information with non-human technological entities (Bickmore et al., 2005; DeVault et al., 2014; Kissinger et al., 1999; Lucas et al., 2014; Yokotani et al., 2018). Given technology’s absence of moral judgement, this may not be a convincing point to make on its own, because the same reasoning would also apply to non-judgmental diaries people write. However, in combination with other empirical findings, one could argue such self-disclosure patterns reflect a more general tendency of humans to differentiate and appreciate certain variations of social engagements according to the perceived strengths and weaknesses of human or non-human engagement partners. For example, Logg et al. (Logg et al., 2019) showed how humans tend to place more trust in predictions and judgements from a technological entity (i.e., algorithm), while, according to Ha et al.’s (Ha et al., 2020) experimental research on privacy concerns and self-disclosure, humans tend to mistrust technological entities which seem too humanlike (e.g., using emotional conversational tones, pro-actively addressing human users), or which present themselves as ‘partners’ rather than ‘servants’.

Similar nuances can also be found in children’s engagements with technological entities. For example, some children in Turkle’s (2017) ethnographic studies provided well-argued reasons why the programmable nature of technology would make them more reliable, consistent and trustworthy than humans. When it comes to DVAs, in particular, the exploratory qualitative findings by Festerling & Siraj (2020) on children’s open engagements with DVAs suggest children seem to appreciate the instant social gratification and excitement they experience with DVAs, and they also associate DVAs with relative ontological strengths. For instance, children systematically attributed higher accuracy levels and faster response times to DVAs for knowledge-related domains of intelligence (e.g., provision of facts), and explained their attribution patterns by DVAs’ connectedness to the internet and their programmable nature (Festerling & Siraj, 2020). This is in line with other empirical findings in the literature on children’s differentiated perceptions of technology as data-based knowledge sources (e.g., (Rücker & Pinkwart, 2016; Wang et al., 2019b)). However, Oranç & Küntay (2020) found even when children think technological entities are intelligent enough to answer questions related to mechanical or electronic issues, for biological and psychological issues (e.g., ‘Why do humans sleep?’, ‘Why do people help each other?’), children still prefer humans as knowledge sources. Similarly, Festerling & Siraj (2020) also found children associate other domains of intelligence with humans (e.g., conversational comprehension, common sense, creativity), which is further in line with Xu et al.’s (2021) experimental findings on how children seem to elevate the intelligibility of their speech according to DVAs’ perceived conversational weaknesses. Lastly, Yip et al.’s (2019) found although children expect a DVA to make them laugh in response to certain commands (e.g., commands to make farting noises), a DVA that would laugh itself was thought of as being utterly disturbing.

In summary, the pragmatistic reading of these empirically observable nuances is that children’s engagements patterns with technological entities already follow nuanced understandings and expectations regarding the entities’ experienceable strengths and weaknesses. This further suggests human-technology engagements are irreducible experiences in their own right on the continuum of social engagements, and, vis-à-vis human-human engagements, the relationship is as much about relative differences as about similarities.

Importantly, although this pragmatistic line of reasoning challenges traditional ethical criticisms which emerge from dualistic viewpoints on reality, it does not negate ethical issues. As Damiano & Dumouchel (Castro-González et al., 2016) show in their account of ‘synthetic ethics’, creating technology inspired by an understanding of, and knowledge about, humanness requires ethical sensitivity towards *concrete* issues which could arise in the context of application, and a solution-oriented attitude to address these issues *without* condemning humanlike technology as a whole. For example, the above discussed use of technology in clinical contexts prompts a question about which humanlike design components could compromise a technology’s usability and effectiveness (see (Bartneck et al., 2010)) which may also be true for other professional contexts (Riether et al., 2012). Furthermore, child-DVA engagements raise additional questions about how DVAs are supposed to handle morally sensitive situations: these include offensive voice commands (UNESCO and EQUALS Skills Coalition, 2019), the close emotional attachments vulnerable children may develop with them (Garg & Sengupta, 2020), and the potential societal harm from using primarily female voice-interfaces (Wang, 2019).

### Pragmatistic View on the *Explanatory* Research Perspective

In a recent experimental study (*n* = 144, age: 8–9 years), van Straten et al. (2020) hypothesized children’s humanlike perceptions of a robot were due to insufficient awareness about the robot’s lack of ‘true’ humanness, as exemplified by its programmability. To address the ambiguity of their empirical findings, van Straten et al. (2020) conclude:

Perhaps it can simply not be assumed that children understand that a humanoid robot is more similar to a technology than to a human being, even if this is pointed out repeatedly and even if children understand that a robot is not humanlike in terms of psychological capacities (p. 12).

But what if researchers’ claims that children anthropomorphize technological entities were simply artefacts of false understandings of what is unique to humans and technology – ‘false’ in the sense it differs from the perspective of today’s children who develop their own understandings of these concepts. In other words, the nature of anthropomorphism could change as humans’ experiential understandings of humanness change, and this may particularly apply to today’s children who are growing up amid technologies which, for example, can emulate the autonomous use of human language and speech. In this case, the claim children anthropomorphize DVAs would be an artefact of an ‘anachronistic’ understanding of humanness and technology – at least from the perspective of children who have been intimately exposed to these voice-enabled technological entities from the beginning of their lives. Or, as put more generally by Damiano and Dumouchel (2018), p.4), with technological progress blurring the experienceable boundaries between humans and technology, “the question of what constitutes human identity, or particularity, is raised anew”. At its core, this is a pragmatistic line of reasoning regarding the explanatory origins of anthropomorphism, because it prioritizes experiential understandings of humanness over a priori derived ones. But what are the supporting arguments for this reasoning?

According to Epley et al.’s (2007) previously mentioned three-factor theory, the process of cognitive inference which causes anthropomorphism psychologically is anchored in the inductive base of humanness (i.e., humans’ deeply ingrained knowledge about the self in particular, and about humans in general). However, could children’s experience of engaging socially with DVAs from the very beginning of life impact the development of this inductive base of humanness? It is beyond the scope of this review to provide a definite proof for this hypothesis, but there are two noteworthy empirical studies that can support this proposition.

First, in a recent neuroscientific study, Waytz et al. (2019) investigated anthropomorphism in amygdala-damaged patients (and a control group) to isolate two neuronal processes: the bottom-up process of anthropomorphism based on overt social behaviors (e.g., facial expressions and motion patterns displayed by a dog), and, in the absence of overt social behaviors, the top-down process based on abstract semantic knowledge about socially meaningful stimuli in the environment (e.g., inferences made from more nuanced social stimuli displayed by a robot). As noted by Waytz et al. (2019), these findings support the general notion that anthropomorphism, as part of social cognition, offers different routes, and that at least one of these routes seems to be based on things humans gradually learn through social experiences as they develop.

Second, in a recent experimental study, Brink et al. (2019) investigated how the ‘uncanny valley’ unfolded in a developmentally diverse sample (*n* = 240, age: 3–18 years). In particular, the study examined whether the uncanny valley constitutes an innate aversion due to evolutionary determined responses to physiological illnesses and defects, which would further imply that even the youngest infants in the sample should display aversions towards overly humanlike technology, or whether it constitutes a violation of postnatally acquired expectations and norms about humanness and technology, which are only developed throughout childhood as part of social learning experiences (Brink et al., 2019). The empirical results revealed an unambiguous pattern: aversions towards overly humanlike technology seem to develop with age, but not before middle childhood (9–11 years), therefore providing strong empirical support for the latter origin of the uncanny valley (Brink et al., 2019).

Taken together, both studies suggest the human stance towards technology is at least partly shaped by children’s developing understandings of the social realities they face. In consequence, this would imply that the inductive base of anthropomorphism, as circumscribed in Epley et al.’s (2007) three-factor theory, could also change through changing social realities. The reality for many children today is both that they can engage with technology through human language and speech, and that these technologies are firmly embedded in social life at home (Ammari et al., 2019; Beirl et al., 2019; Porcheron et al., 2018; Voit et al., 2020; Garg & Sengupta, 2020; Lee et al., 2020).

This pragmatistic view on anthropomorphism echoes previous discussions in the literature on Kahn et al.’s (2006, 2007, 2011, 2009, 2012b) ‘new ontological category hypothesis’, stating increasingly intelligent technologies could become an independent and developmentally stable ontological category in its own right which cuts across traditional ontological dichotomies (e.g., animate vs. inanimate, human vs. non-human). Implications, challenges and potential empirical validations of the new ontological category hypothesis have already been discussed elsewhere (e.g., (van Straten et al., 2020; Severson & Lemm, 2016; Severson & Carlson, 2010; de Graaf, 2016; Seibt, 2017; Oranç & Küntay, 2020; Levillain & Zibetti, 2017; Melson et al., 2009; Bernstein & Crowley, 2008; Gaudiello et al., 2015; Jipson et al., 2016; Kim et al., 2019)), and also in the particular context of DVAs (e.g., (Festerling & Siraj, 2020; Pradhan et al., 2019)). However, this review adds that the new ontological category hypothesis is philosophically grounded in a pragmatistic paradigm, because the underlying assumption is that children’s developing ontologies, as reflected by their conceptual understandings of the world around them, may not converge to an a priori definable and metaphysically ‘true’ end-state. In the words of James (1912/2012, p.114): “[o]ur ideas and concepts and scientific theories pass for true only so far as they harmoniously lead back to the world of sense”.

## Conclusion, Limitations, and Future Research

This conceptual review article, addressed to researchers interested in anthropomorphism and adjacent areas related to human-technology or child-technology engagements, has aimed to provide a conceptually framed account of contemporary anthropomorphism research based on three different research perspectives (descriptive, normative, and explanatory). Moreover, it has applied a pragmatistic viewpoint (mainly inspired by Jamesian pragmatism and related works) to discuss how these perspectives may contribute to a scientific understanding of children’s engagements with increasingly intelligent and omnipresent technologies such as DVAs. The pragmatistic viewpoint sheds a new light on widely held views in the literature (e.g., descriptive ‘as if’ claims, normative hierarchies between human-human and human-technology engagements), culminating in the argument that, from an explanatory perspective, the nature of anthropomorphism may change as humans’ experiential understandings of humanness change, and that this may particularly apply to today’s children as their social cognition develops in interaction with technological entities which are increasingly characterized by unprecedented combinations of human and non-human qualities.

Besides the methodological limitations inherent to conceptual reviews (Parsons, 2016), the article’s main limitation lies in the trade-off choice between analytical scope and depth: to increase the scope of the discussion, the article referred to ‘technology’ as a rather broad category comprising various types of technological entities and social engagements (see research overview in supplementary material), while special attention was only paid to the peculiarities of children’s engagements with DVAs. Narrowing the scope to certain types of technology or social engagements would have partly increased the depth of the discussion. However, the aim of this article was to look at the bigger picture of anthropomorphism research investigating humans’ engagements with technology, in general, and children’s engagements with technology, in particular. As such, the question whether the technology under investigation is specifically designed for social purposes is not of primary importance, because, as the above discussion suggests, there are infinitesimal variations of how technology can be anthropomorphized, and these occur across a broad range of design and research contexts.

The article points in three main directions for future research. First, recent empirical studies which investigated traditional anthropomorphism issues in human-technology research from a developmental perspective (e.g., developmental origins of the uncanny valley by Brink et al. (Brink et al., 2019)) already showed how the investigation of children can contribute to the research field more generally. Future research could use similar cross-sectional or experimental age group comparisons to investigate how children’s daily exposure to certain types of technology (e.g., DVAs) may influence such developments. For instance, Bernstein & Crowley’s (Bernstein & Crowley, 2008) original, and, up to this point, un-replicated finding that children born in the early 2000s with high exposure to robotic technologies have a different conceptual understanding of intelligence and aliveness raises overdue empirical questions in light of hundreds of millions of DVAs being installed in home and childhood environments across the globe in the early 2020s. Following the pragmatistic viewpoint of the above discussion, such future research should be less concerned with the question whether children develop an ‘objectively true’ understanding of technology (see (van Straten et al., 2020)) and more open towards new ways about thinking about their increasingly technologized environments (Festerling & Siraj, 2020).

Second, research on children’s understandings of state-of-the-art technology is trying to capture a fast-moving target, especially in the context of commercially available technologies such as DVAs. Unlike many other types of technologies often investigated in anthropomorphism research, DVAs not only stand out in terms of how fast they have populated home and childhood environments at large scale but also how fast their designs and technological capacities to emulate human language and speech change. Therefore, DVAs remain a highly relevant yet challenging case for research in their own right, and particular attention should be paid its technological development. For instance, so far DVAs’ human-like design has been limited to the autonomous use of human language and speech, but smart speakers could soon also be able to move autonomously (e.g., turning screens according to user movements in the room) (Amazon Day One Staff, 2020). The utilitarian importance of such technological developments may be unclear, but given coherent motion patterns are a salient stimulus for innate social cognition in their own right (e.g., (Meltzoff et al., 2010; Bertenthal et al., 1987; Simion et al., 2008)), they could be important in children’s developing understandings of DVAs. In this context, children with disabilities and their caregivers will be a subpopulation of particular importance, given DVAs’ (future) potential to *inform*, *assist*, *assess* and *support* these individuals with special developmental or medical needs (Sezgin et al., 2020). Preliminary research already suggests commercial off-the-shelf DVAs are popular among individuals with disabilities (e.g., (Pradhan et al., 2018; Duffy et al., 2021)), but research is still limited, especially when focusing on concrete disabilities (e.g., (Cave & Bloch, 2021)). However, apart from general socio-economic and skill-related accessibility issues (Paul et al., 2021), children with different kinds of disabilities will experience different kinds of benefits and challenges. For instance, visually impaired children are likely to benefit from the functional advantage of DVAs’ hands-free and ‘eye-free’ voice interfaces, but, at the same time, today’s DVAs usually lack the customizability to address certain special needs, such as longer response times, faster speech rates, or custom voice commands (Branham & Mukkath Roy, 2019). Nevertheless, future research should not only consider DVAs as a functional means to a social end for children with disabilities (e.g., by using text-to-speech functions to socially connect with peers online, see (Paul et al., 2021)), but also as a social end in itself (e.g., by serving as a humanlike ‘scaffold’ to improve social cognition, see (Atherton & Cross, 2018)).

Third, the pragmatistic view on anthropomorphism can be extended by what neo-pragmatists (e.g., (Rorty, 1982, 1998, 2006)) say about the critical role of language in inquiry. In fact, human cognition is significantly shaped by the languages spoken (Haun et al., 2006), and in the context of anthropomorphism, determining the ‘true’ meaning of what humans say about or to non-human entities remains challenging from a research perspective (Damiano & Dumouchel, 2018; Złotowski et al., 2015). For example, the use of gendered personal pronouns in human-DVA engagements has been interpreted as a direct signifier for the personalization of DVAs (e.g., (Gao et al., 2018; Pradhan et al., 2019; Purington et al., 2017)). Future research could be more critical of the role of language in anthropomorphism by examining the psychological mechanisms (e.g., sociality or effectance motivation) which cause the development of particular language patterns in human-technology engagements (e.g., referring to DVAs with personal pronouns).

Lastly, it is worth pointing out that our discussion is ‘bent towards a particular perception of science’. In particular, the notion of pragmatism we follow lends itself to an empirically grounded perspective to look at things, that is, a perspective which favors “a [constructivist] model of children as builders of their own intellectual structures” (Papert, 1980, p.7), rather than a model of children as recipients of a priori defined intellectual structures we already built for them. Developmental constructivism – at least in its original epistemological form – has traditionally been criticized for not having a socio-cultural lens (Chapman, 1988), and readers who feel more inclined towards this criticism will naturally entertain doubts about the ideas and arguments discussed above. Yet, we hope our paper widens the thinking on children’s development in the context of modern everyday technology, and how social cognition of today’s children develops in interaction with technological entities which are increasingly characterized by unprecedented combinations of human and non-human qualities.

## Supplementary Information

ESM 1 (DOCX 120 kb)

## Footnotes

1 The review does not differentiate between *anthropomorphism* and *animism*. While the latter term refers more generally to cases in which humans engage with *non-living* entities as if they were *alive*, the former term refers only to those cases in which humans engage with *non-human* entities as if they were *human (*Guthrie, 1993*)*. In addition, this review mainly focuses on anthropomorphism in the context of technology, which differs from anthropomorphism in other contexts (e.g., anthropomorphism in the ethological context of animal behavior) (Severson & Woodard, 2018). Therefore, the review acknowledges any references to other contexts must be treated with caution.

2 For this reason, we also find terms such as ‘unembodied AI’ problematic, because embodiment may rarely be determinable on a simple ‘yes’ or ‘no’ basis.

3 In contrast to Servais’ (2018) pragmatistic account of anthropomorphism research, which argues an entity’s inner state (e.g., intentions, feelings) is *directly* perceivable without the need for inference (e.g., (Gallagher, 2008)), this review’s pragmatistic view on anthropomorphism research commits to the ‘anthropomorphism-as-inference’ approach, such as Epley et al.’s (2007) three-factor theory.
