# Supplementary material for: Anthropomorphizing Technology: A Conceptual Review of Anthropomorphism Research and How it Relates to Children’s Engagements with Digital Voice Assistants
Source: Integr Psychol Behav Sci. 2021 Nov 23;56(3):709–38. doi: 10.1007/s12124-021-09668-y (PMC9334403; doi:10.1007/s12124-021-09668-y)
Supplement: Supplementary file 1 — (DOCX 120 kb) [file 12124_2021_9668_MOESM1_ESM.docx]

| **Supplementary Material.** Overview of empirical research on human-technology engagements | | | | |
| --- | --- | --- | --- | --- |
| *Human-technology research* | | | | |
| Reference | Sample and age group | Type of technology | Type of social engagement | |
| Bartneck & Hu [62] | *n*=20 (university students) | Non-humanoid robot (*Microbug*) | Refraining from abusive behaviors towards technology | |
| Bartneck et al. [175] | *n*=44 (university students) | Animalistic (*iCat*) and non-animalistic robots (industrial robot) | Feelings of embarrassment towards technology depending on its animal-likeness | |
| Castro-González et al. [96] | *n*=42 (18 to 58 years) | Robots with different degrees of human-likeness | Perception of technology’s likability depending on how naturalistic/ humanlike its movements are | |
| de Visser et al. [63] | *n_1_* = 37* (university students) | Computer screen displaying humanoid avatar | Perceptions of technology’s trustworthiness | |
| DeVault et al.  [166] | *n*=91 (young adults) | Computer screen displaying  humanoid avatar (either with or without social-emotional functions) | Self-disclosure of personal information to technology (depending on its social-emotional functions) | |
| DiSalvo et al. [59] | *n*=40 (adults) | 48 robots (incl. research robots, commercially available robots, fictional robots) | Perceptions of technology’s human-likeness and likability | |
| Evers et al. [71] | *n*=135 (young adults) | Non-humanoid robot | Perceptions of technology’s human-likeness and trustworthiness (depending on human’s socio-cultural background and sense of control) | |
| Eyssel & Reich [73] | *n*=34 (university students) | Humanoid robot (*FloBi*) | Perceptions of technology’s human-likeness (depending on human’s feeling of loneliness) | |
| Eyssel et al. [66] | *n*=31 (university students) | Animalistic robot (*iCat*) | Perceptions of technology’s likability, closeness and pleasantness | |
| Eyssel et al. [65] | *n* = 58 (university students) | Humanoid robot (*FloBi*) with either humanoid or robotic voices | Perceptions of technology’s human-likeness, likability, and closeness (depending on human’s gender in relation to technology’s embodied gender) | |
| Ferrari et al. [119] | *n_1_*=182 (19 to 63 years)  *n_2_*=51 (university students) | Robots with different degrees of human-likeness | Perceptions of technology’s threatening nature (depending on its human-likeness) | |
| Goetz et al. [103] | *n*=176* (university students) | Humanoid robot (*Nursebot Pearl*) | Human’s acceptance of and cooperation with technology (depending on its appearance and task-oriented behavior) | |
| Goudey & Bonnin [72] | *n*=172 (female adults 25 to 55 years) | Robots with different degrees of human-likeness (*Nao*, *Emox*, and *PaPeR*o) | Perceptions of technology’s likability and the role of the perceiver’s prior experience with technology | |
| Ha et al. [171] | *n*=296* (adults) | DVA (*Alexa, Siri* and *Google Assistant*) | Self-disclosure of personal information to technology (depending on perceptions of technology’s human-likeness) | |
| Kahn, Severson et al. [182] | *n*=40 (university students) | Humanoid robot (*Robovie*) | Perceptions of technology’s moral accountability | |
| Kamide et al. [70] | *n*=3,543* (15 to 79 years) | 11 humanoid robots (incl. *Robovie, ASIMO* and *Geminoid*) | Perceptions of technology’s human-likeness (depending on human’s gender and age) | |
| Kim & McGill [74] | *n*=224* (university students) | Slot machine with different degrees of human-likeness | Perceptions of technology’s human-likeness and agency (depending on human’s risk-aversion and feelings of control) | |
| Logg et al. [170] | *n*=671* (adults) | Information generated by algorithms | Trusting behaviors towards technology | |
| Lucas et al. [168] | *n*=239 (18 to 65 years) | Computer screen displaying  humanoid avatar | Self-disclosure of personal information to technology | |
| Mutlu et al. [61] | *n*=26 (university students) | Robots with different degrees of human-likeness (*Gemenoid* and *Robovie*) | Recognizing signals of intention (e.g., gazing) displayed by technology | |
| Nass et al. [55]–[57] | *n* = 194* (university students) | Personal computers (incl. desktop computers using simple voice-outputs) | Adopting friendly and polite behaviors towards technology | |
| Riether et al. [104] | *n*=106 (university students) | Humanoid robot (*FloBi*) | Technology’s social facilitation effect on humans’ task solving performance | |
| Shah et al. [98] | *n*=16 (adults) | Humanoid robot (*Nexi*) | Effectiveness of human-technology collaboration (depending on technology’s human-like teamwork behaviors) | |
| Walters et al. [60] | *n*=79 (university students) | Robots with either humanoid or non-humanoid appearances | Perceptions of technology’s human-likeness and likability (depending on human’s personality, such as introversion and emotional stability) | |
| Wang [46] | *n*=373 (university students) | Smartphones | Perceptions of technology’s human-likeness (depending on human’s feeling of loneliness, attachment style and socio-cultural background) | |
| Waytz et al. [68] | *n*=957* (university students) | Miscellaneous | Perceptions of technology’s consciousness, free will, intentions, mindedness and emotions | |
| Waytz, Morewedge et al. [75] | *n*=301* (university students and adult museum visitors) | Personal computers, robotic gadgets, and humanoid robot (*ASIMO*) | Perceptions of technology’s human-likeness (depending on predictability of its behavior and human’s effectance motivation to predict its behavior) | |
| Yokotani et al. [169] | *n*=98 (university students) | Computer screen displaying  humanoid avatar | Self-disclosure of sensitive personal information to technology | |
| Złotowski et al. [99] | *n*=35 (university students) | Humanoid robot (*Nao*) | Perception of technology’s human-likeness (depending on its observable emotionality and intelligence) | |
| *Child-technology research (incl. DVA research)* | | | | |
| Reference | Sample and age group | Type of technology | Type of social engagement | |
| Beirl et al. [4] | *n_1_*=10 (adults)  *n_2_*=9 (6 to 11 years) | DVAs (*Alexa, Google Assistant*) | Social-behavioral routines with technology within the home environment | |
| Brink et al. [110] | *n*=240 (3 to 18 years) | Robots with different degrees of human-likeness (*Kaspar* and *Nao*) | Perceptions of technology’s likability/creepiness and human-likeness | |
| Festerling & Siraj [10] | *n*=27 (6 to 10 years) | DVAs (*Alexa* and *Google Assistant*) | Perceptions of technology’s humanoid and non-humanoid ontological nature | |
| Garg & Sengupta [12] | *n_1_*=34 (30 to 65 years)  *n_2_*=25 (5 to 15 years) | DVAs (*Google Assistant*) | Long-term social-behavioral routines with technology within the home environment | |
| Meltzoff et al. [130] | *n*=64 (18-month-old infants) | Humanoid robot (*HOAP*) | Recognizing signals of intention (e.g., gazing) displayed by technology | |
| Oranç & Küntay [174] | *n*=80 (3 to 6 years) | Humanoid robot (*Nao*) | Trusting behaviors towards technology | |
| Porcheron et al. [5] | *n_1_*=10 (adults)  *n_2_*=4 (children) | DVA (*Alexa*) | Social-behavioral routines with technology within the home environment | |
| Severson & Lemm [80] | *n_1_*=654* (university students)  *n_3_*=90 (5 to 9 years) | Miscellaneous | Perceptions of technology’s consciousness, free will, intentions, mindedness and emotions | |
| Severson & Woodard [79] | *n*=90 (5 to 9 years) | Miscellaneous | Perceptions of technology’s consciousness, free will, intentions, mindedness and emotions (depending on human’s tendency for imagination) | |
| van Straten et al. [45] | *n*=144 (8 to 9 years) | Humanoid robot (*Nao*) | Perceptions of technology’s human-likeness and trustworthiness | |
| Wang et al. [173] | *n*=60* (5 to 8 years) | Information from an unspecified technological source | Trusting behaviors towards technology | |
| Woods [111] | *n*=159 (9 to 11 years) | 40 robots (incl. humanoid and animalistic robots and non-humanoid robots) | Perceptions of technology’s likability/creepiness and human-likeness | |
| Xu et al. [109] | *n*=90 (3 to 6 years) | DVA (*Google Assistant*) | Effectiveness of technology as an interactive learning partner for reading activities | |
| Yip et al. [112] | *n*=11 (7 to 11 years) | Miscellaneous | Perceptions of technology’s likability/creepiness | |
| *DVA research (other)* | | | | |
| Reference | Sample and age group | Type of technology | | Type of social engagement |
| Ammari et al. [3] | *n*=19 (19 to 63) | DVAs (*Alexa, Google Assistant, Siri*) | Social-behavioral routines with technology within the home environment | |
| Chin et al. [67] | *n*=94 (university students) | DVA (*Google Assistant*) | Feelings of guilt towards technology displaying empathetic response behaviors | |
| Lee et al. [13] | *n*=218 (adults) | DVAs (*Alexa, Google Assistant, Siri*) | Perceptions of technology’s facilitation of social dynamics and group harmony within the home environment | |
| Purington et al. [50] | *n*=587 (customer reviews) | DVA (*Alexa*) | Perceptions of technology’s likability; attributions of social roles to technology (e.g., friend, family member); the role of the perceiver’s personification (e.g., referring to technology with personal pronouns) | |
| Voit et al. [7] | *n*= 17 (21 to 35 years) | DVA (*Alexa*) | Social-behavioral routines with technology within the home environment | |
| *Notes.* Table provides an overview of empirical research on human-technology engagements reviewed in the article. Within categories, references are listed in alphabetical order. For some references, not all studies included in the original publication are mentioned in the table. * indicates that the sample size is an aggregate of different studies with similar participants. | | | | |
